# Endoplasmic Reticulum Detergent-Resistant Membranes Accommodate Hepatitis C Virus Proteins for Viral Assembly

**DOI:** 10.3390/cells8050487

**Published:** 2019-05-22

**Authors:** Audrey Boyer, Julie Dreneau, Amélie Dumans, Julien Burlaud-Gaillard, Anne Bull-Maurer, Philippe Roingeard, Jean-Christophe Meunier

**Affiliations:** 1 INSERM U1259, Université de Tours et CHRU de Tours, 37032 Tours, France; Audrey.Boyer@live.fr (A.B.); julie.dreneau@etu.univ-tours.fr (J.D.); amelie.dumans@univ-tours.fr (A.D.); julien.gaillard@univ-tours.fr (J.B.-G.); anne.bull@univ-tours.fr (A.B.-M.); roingeard@med.univ-tours.fr (P.R.); 2 Plate-Forme IBiSA des Microscopies, PPF ASB, CHRU de Tours, 37032 Tours, France

**Keywords:** HCV, morphogenesis, detergent resistant membrane, Apolipoprotein E (ApoE), NS2

## Abstract

During Hepatitis C virus (HCV) morphogenesis, the non-structural protein 2 (NS2) brings the envelope proteins 1 and 2 (E1, E2), NS3, and NS5A together to form a complex at the endoplasmic reticulum (ER) membrane, initiating HCV assembly. The nature of the interactions in this complex is unclear, but replication complex and structural proteins have been shown to be associated with cellular membrane structures called detergent-resistant membranes (DRMs). We investigated the role of DRMs in NS2 complex formation, using a lysis buffer combining Triton and n-octyl glucoside, which solubilized both cell membranes and DRMs. When this lysis buffer was used on HCV-infected cells and the resulting lysates were subjected to flotation gradient centrifugation, all viral proteins and DRM-resident proteins were found in soluble protein fractions. Immunoprecipitation assays demonstrated direct protein–protein interactions between NS2 and E2 and E1 proteins, and an association of NS2 with NS3 through DRMs. The well-folded E1E2 complex and NS5A were not associated, instead interacting separately with the NS2-E1-E2-NS3 complex through less stable DRMs. Core was also associated with NS2 and the E1E2 complex through these unstable DRMs. We suggest that DRMs carrying this NS2-E1-E2-NS3-4A-NS5A-core complex may play a central role in HCV assembly initiation, potentially as an assembly platform.

## 1. Introduction

Hepatitis C virus (HCV) circulates as hybrid particles in patients. These lipoviral particles (LVPs) combine components of HCV and very low-density lipoprotein (VLDL) [1,2,3,4]. However, the mechanisms of LVP morphogenesis and the nature of the assembly mechanisms remain poorly understood. Several recent studies have reported an essential role for nonstructural protein 2 (NS2) early in HCV morphogenesis [5,6,7,8,9]. This protein is proposed to organize the coalescence of viral components at the particle assembly site. Together with p7, HCV NS2 recruits the envelope glycoproteins E1, E2, and NS3 to a site close to the replication complex (RC) at the endoplasmic reticulum (ER) membranes. It remains unclear whether NS5A is a component of this interaction. Several studies have reported NS5A to be present in this large complex [6,8]. By contrast, others have reported an absence of association between NS5A and NS2 [9]. Ma et al. suggested that NS2 forms two different complexes, one with E1, E2, and NS3, and the other with NS5A [7]. Nevertheless, the mechanism associating these partners in assembly remains unclear.

Several groups have reported that mutations affecting transmembrane domains of p7, E1, E2, or NS2 might disrupt the formation of this large complex organized by NS2 [6,8,9,10]. The association of these viral proteins with membranes may therefore be important for complex formation. In addition, several studies have suggested that these proteins are associated with specific areas of the ER membrane. Using a subgenomic replicon, Tedbury et al. demonstrated that NS2 localization was influenced by the presence of the upstream p7, with its cognate signal peptide derived from the C terminus of E2 (SPp7). NS2 is found in the detergent-insoluble fractions of cellular membranes [11], and it has recently been shown than an association of NS2 and E2 with detergent-resistant membranes (DRMs) is required for efficient viral assembly [12]. The structural proteins and RC have also been reported to associate with DRMs [13,14,15,16]. These structures are observed in low-density fractions of the flotation gradient after cell lysis with non-ionic detergents (such as Triton X-100) in cold conditions. Lipid rafts are the physiological structures of cells corresponding to DRMs, as they are resistant to extraction with non-ionic detergents. Lipid rafts are cellular-membrane-specific domains enriched with glycosphingolipids and cholesterol. They are enriched with resident proteins (such as flotillin) and lipid-modified proteins (containing saturated acyl chains) [17]. Several viruses have been reported to use lipid rafts for their own assembly and budding, and these domains may contain all the proteins required for viral morphogenesis [18]. Lipid rafts have generally been described in the plasma membrane, but they have also been identified in ER membranes [19,20].

We investigated the role of DRMs in interactions between NS2, NS3, NS5A, E1, E2, and core during assembly, using a strategy involving the efficient disruption of DRMs to identify interactions between the proteins they contained. We also used the AR3A and ARA5 human monoclonal Abs (antibodies) isolated by Dr. Law’s group [21], which target different antigenic regions (ARs). The AR3A epitope is conserved, and AR3A binds to E2 only. This Ab recognizes E2 alone or in association with E1 (AR3A-E2 species). The AR3A epitope is conformational, but does not require correct folding of the E1E2 complex. It therefore recognizes partially or correctly folded monomeric E2 species and partially or correctly folded E1E2 complexes. The AR5A binding site is present on the E1E2 complex and requires E1. Correct folding of the E1E2 complex is essential for AR5A binding (AR5A-E1E2 species). Using these Abs, we assessed the relationship between the folding step reached by envelope proteins and the viral and cellular proteins with which they interacted. Our goal was to characterize a protein coalescence process organized around DRMs by NS2 and initiating viral assembly.

Our results demonstrate the occurrence of a direct (protein–protein) interaction between NS2 and AR3A-E2 species. By contrast, the well-folded AR5A-binding E1E2 complex was associated with NS2 via a specific lipid environment, the DRM. Interestingly, the association of NS2 with well-folded E1E2 complexes was much weaker than that with AR3A-E2 species. Furthermore, NS2, NS3, NS5A, and core did not interact directly with each other, but all interacted with DRMs. Our study suggests that, in physiological conditions, DRMs (lipid-raft-like structures) associated with E1E2 glycoproteins, NS2, NS3, NS5A, and core may act as assembly platforms for HCV particles.

## 2. Materials and Methods

### 2.1. Cells and Cell Lysis

We used Huh7.5 cells (kindly provided by Charles Rice, Rockefeller University, New York, NY, USA) carrying the full-length JFH1 genome, including a gene encoding NS2 with an HA tag, and the anti-E1 Ab A4 epitope was introduced by reverse engineering (A4HAHCVcc; kindly provided by Jean Dubuisson, Pasteur Institute of Lille, Lille, France [8]). Cells were lysed with Tx buffer (1% Triton X-100, 2 mM EDTA, protease inhibitor cocktail in phosphate-buffered saline (PBS)) or with Tx buffer + freeze–thaw + n-octyl buffer (60 mM n-octyl-b-d-glucopyranoside, 2 mM EDTA, protease inhibitor cocktail in phosphate-buffered saline (PBS)): cells were first incubated with Tx buffer for 1 h at 4 °C. The fragments were collected as a pellet after centrifugation, and the supernatant was frozen overnight or for longer. The lysate was thawed and treated with Tx or n-Octyl buffer (equal volumes). We also used lysate with Tx buffer but without freezing.

### 2.2. Antibodies

The human monoclonal anti-E2 Ab (AR3A) and the human monoclonal anti-E1E2 complex Abs (AR4A and AR5A) [21] were kindly provided by Mansun Law (SCRIPPS, California, CA, USA). The rat monoclonal anti-E2 Ab (3/11) was kindly provided by Jane McKeating (University of Birmingham, Birmingham, UK). The mouse monoclonal anti-E1 Ab (A4) [22] was kindly provided by Harry Greenberg (Stanford University, California, USA). The mouse monoclonal anti-NS5A Ab (9E10) was kindly provided by Charles Rice (The Rockefeller University, New York, NY, USA).

The following antibodies were purchased: mouse monoclonal anti-core Ab (C7-50: MA1-080, Thermo Fisher Scientific, Courtaboeuf, France), mouse monoclonal anti-NS3 Ab (8G-2: ab65407, Abcam, Paris, France), mouse monoclonal anti-HA Ab (16B12, Covance, Paris, France), rabbit monoclonal anti-HA (ab9110, Abcam), mouse monoclonal anti-flotillin-1 Ab (BD Biosciences, San Jose, CA, USA), rabbit polyclonal anti-calnexin Ab (Santa Cruz Biotechnology Inc., Dallas, TX, USA), and IgG isotypic controls from R&D Systems: mouse monoclonal IgG1, purified human IgG, and rabbit polyclonal IgG.

### 2.3. Membrane Fractionation

Membranes were fractionated as previously described [23]. We mixed 2 ml of clarified cell lysate with an equal volume of 90% sucrose in 2-(*N*-morpholino)ethanesulfonic acid (MES)-buffered saline (25 mM MES, 150 mM NaCl, 2 mM EDTA, pH 6.5) to generate a 45% sucrose solution. A discontinuous sucrose gradient was created by layering 4 ml each of 35% and 5% sucrose solutions onto the 45% sucrose solution (MES-buffered saline with 250 mM Na_2_CO_3_). Gradients were centrifuged (ThermoFisher scientific, Courtaboeuf, France, Ultracentrifuge Sorvall WX 90+) for 17 h at 280,000× *g* and 4 °C, and 11 fractions (1 mL each) were collected. Flotillin was used as a marker for the identification of DRM fractions, and calnexin was used as a marker for soluble fractions. Both markers were assessed by western blotting. Equal volumes of the various fractions were loaded onto the gel.

### 2.4. Immunoprecipitation Assay

Clarified cell lysates were incubated with a slurry of Sepharose beads (rec-Protein G-Sepharose^®^ 4B Conjugate, Invitrogen, Courtaboeuf, France) conjugated with antibodies in PBS. Incubation conditions were kept homogeneous by adding n-octyl-b-d-glucopyranoside during immunoprecipitations of lysate with Tx buffer. The beads were washed four times in PBS–0.1% Triton X-100 and the immunocomplexes obtained were analyzed by western blotting. Samples were resolved by SDS-polyacrylamide gel electrophoresis (PAGE 12%), and the bands obtained were transferred electrophoretically onto PVDF membranes with a Trans-Blot apparatus (Biorad, Marnes-la-coquette, France). The membrane was then probed with anti-E2, anti-E1, anti-HA, anti-NS5A, and anti-NS3 antibodies, followed by a peroxidase-conjugated secondary antibody. Labeled antibodies were detected by enhanced chemiluminescence (ECL), according to the procedure recommended by the kit manufacturer. HCV core was quantified in a fully automated microparticle chemiluminescence immunoassay (Architect HCV Ag; Abbot, Chicago, IL, USA).

### 2.5. Electron Microscopy Analysis of the Ultrastructure of the Infected Cells

Huh7.5 cells were infected with JFH1-HA-A4, fixed three days post-infection by incubation for 30 min with 4% paraformaldehyde in phosphate buffer (pH 7.6), and washed with PBS. Cell pellets were embedded in 12% gelatin and infused with 2.3 M sucrose for 2 h at 4 °C. We cut 90 nm ultrathin cryosections at −110 °C on a LEICA UCT cryo-ultramicrotome. The sections were retrieved in a 2% methylcellulose/2.3 M sucrose mixture (1:1) and collected on formvar/carbon-coated nickel grids. The sections were saturated by incubation with 1% BSA in PBS and incubated for 1 h with a 1:50 dilution of antibody in PBS. The grids were washed six times and incubated with 10 nm and 6 nm gold particles conjugated directly to antibodies diluted 1:30 in PBS. Finally, the grids were washed, post-fixed in 1% glutaraldehyde, and rinsed in distilled water. The sections were contrast-stained with a mixture of 4% uranyl acetate and 2% methylcellulose (1:10 mixture). The sections were imaged in a transmission electron microscope at 100 kV (JEOL 1011, Tokyo, Japan).

## 3. Results

### 3.1. DRMs Can Be Solubilized in A Combination of Triton X-100 and n-Octyl-β-d-glucopyranoside

**Figure 1:**
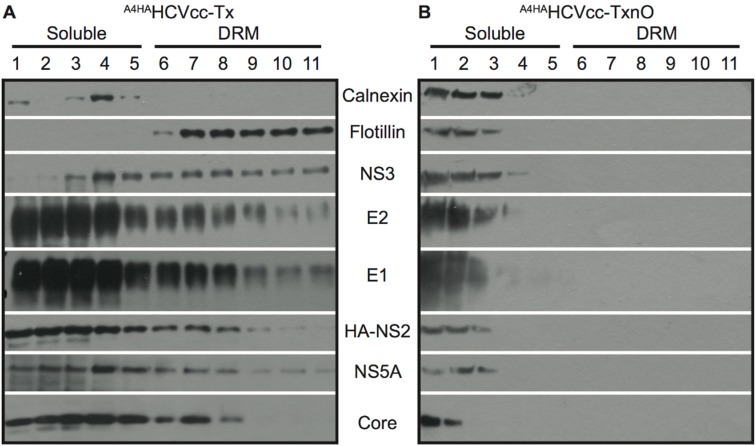
Detergent-resistant membranes (DRMs) are preserved after cell lysis with Triton X-100 (Tx) buffer, but are solubilized by n-octyl-β-d-glucopyranoside buffer (TxnO) buffer. Membrane flotation assay of (**A**) ^A4HA^HCVcc-infected cells lysed with Triton X-100 (Tx) buffer and (**B**) ^A4HA^HCVcc-infected cells lysed with Tx buffer then frozen/thawed and treated with n-octyl-β-d-glucopyranoside buffer (TxnO). Each fraction of both gradients was assessed by immunoblotting with antibodies against flotillin, calnexin, E1, E2, NS3, NS5A, HA (detecting NS2), and core. Flotillin was used as a marker for the identification of detergent-resistant membrane (DRM) fractions, and calnexin was used as a marker for soluble fractions. The flotation assays presented here are representative of multiple experiments (≥3) showing similar results.

NS2 recruits the viral proteins involved in initiating nucleocapsid (NC) translocation to the ER. These proteins (glycoproteins E1, E2, and NS3) seem to collect together on the assembly platform, but the mechanism by which this is achieved, and the nature of the assembly platform, remain unclear. Several studies have suggested that lipid rafts (resistant to non-ionic detergents, i.e., DRMs) act as the assembly platform for HCV and other viruses [12,18]. We investigated the involvement of DRMs in the NS2-driven recruitment of viral proteins, with a strategy based on the disruption of these domains, designed to identify the mechanisms of interaction between NS2 and E1, E2, NS3, NS5A, and core proteins. We used two different lysis buffers to produce JFH1-A4-HA-expressing Huh7.5 (A4HA-HCVcc) cell lysates. We optimized DRM solubilization by freeze–thawing samples obtained after lysis in Tx buffer (TxF) before incubating them with n-octyl glucopyranoside (TxnO). TxnO completely solubilized the cell membranes and DRMs (as described in Materials and Methods). The lysates were subjected to non-continuous flotation-gradient fractionation, as previously described [23]. Flotillin-I was used as a marker for the identification of DRM fractions, and calnexin was used as a marker of soluble fractions (Figure 1). Following lysis in Tx buffer, HCV E1, E2, NS3, NS2, NS5A, and core were detected in the same fractions as DRMs (i.e., fractions 6 to 11), as previously observed (Figure 1A) [12,13,14]. However, following lysis in TxnO lysis buffer, flotillin was found in the uppermost fractions (soluble proteins), consistent with the complete solubilization of DRMs. HCV proteins were also found in these uppermost fractions (Figure 1B). The results obtained after TxF lysis followed by flotation-gradient fractionation were similar to those obtained after Tx lysis. Thus, after lysis in Tx buffer, significant proportions of HCV E1E2, NS2, NS3, NS5A, and core proteins were detected in the same fractions as proteins resident in DRMs, such as flotillin. In addition, DRMs were fully solubilized in TxnO lysis buffer, with the release of all DRM-associated proteins.

### 3.2. The Tx, TxF, and TxnO Buffers Do Not Alter Specific Protein–Protein Interactions

**Figure 2:**
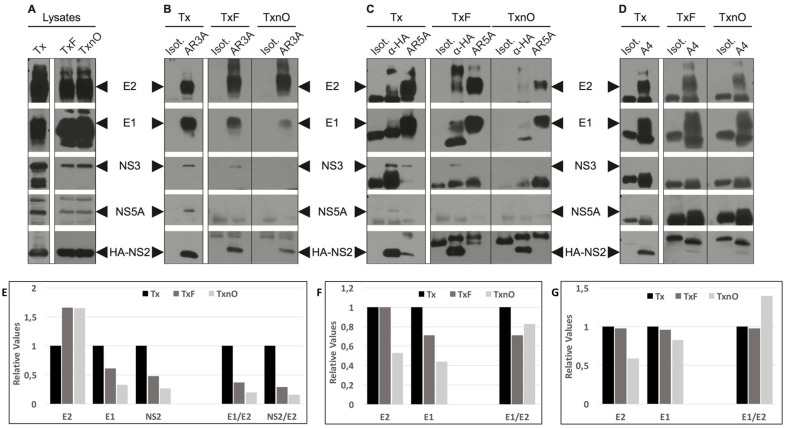
Co-immunoprecipitation assays suggest that HCV proteins are associated with DRMs. ^A4HA^HCVcc-infected cells were lysed with Triton X-100 (Tx) buffer, with Triton X-100 buffer + freezing (TxF), or Triton X-100 buffer + freezing + n-octyl-β-d-glucopyranoside buffer (TxnO). The western blots presented here are representative of multiple experiments (≥3) showing similar results. (**A**) Whole cell lysates were examined by immunoblotting with antibodies against E1, E2, NS3, NS5A, and HA. (**B**) These cell lysates were subjected to immunoprecipitation (Ip) with anti-E2 antibody (AR3A), (**C**) anti-HA (HA-NS2) or anti-E1E2 antibody (AR5A), or (**D**) anti-E1 antibody (A4) and isotypic IgG. The immunoprecipitated proteins were investigated by immunoblotting with anti-E1, anti-E2, anti-NS3, anti-NS5A, and anti-HA (NS2) antibodies. (**E**–**G**) Protein bands were quantified by densitometry using ImageJ software. (**E**) Bands corresponding to E1, E2, and NS2 from AR3A Ip (**B**) were quantified. Tx AR3A Ip band relative density is 1. (**F**) Bands corresponding to E1 and E2 from AR5A Ip (**C**) were quantified. Tx AR5A Ip band relative density is 1. (**G**) Bands corresponding to E1 and E2 from A4 Ip were quantified. Tx A4 Ip band relative density is 1. E1/E2 or NS2/E2: E1 to E2 or NS2 to E2 value ratio, respectively.

We first checked that lysis in different buffers preserved the conformation of the proteins and the interactions between them. We optimized DRM solubilization by freeze–thawing samples obtained after lysis in Tx buffer (TxF), before incubating them with n-octyl glucopyranosyde (as described in Materials and Methods). TxnO is a highly lytic buffer that could potentially alter protein–protein interactions (regardless of DRMs), which would constitute a bias in our tests. It has already been shown that TxnO prevents protein aggregation and can be used to prevent protein denaturation [24]. We nevertheless investigated the association between the E1 and E2 glycoproteins, which have been shown to be strongly associated within cells, forming both non-covalent and covalently associated complexes [25,26]. After lysis with either strategy, immunoprecipitation was performed with the conformational antibody AR5A (targeting well-folded E1E2 complexes) [21] or the linear anti-E1 Ab A4. On co-immunoprecipitation, the E1/E2 ratio was similar for the Tx, TxF, and TxnO strategies when the AR5A and A4 Ab were used (Figure 2F,G), demonstrating that even the most stringent lysis buffer used here did not disrupt protein–protein interactions. Nevertheless, the amount of E1 co-precipitated with AR3A (Figure 2E) was affected by the buffer used, with particularly small amounts obtained when the TxnO buffer was used. Thus, with AR3A, the TxnO buffer may have revealed the presence of protein interactions mediated by membrane lipids, or weak protein–protein interactions, in some cases.

### 3.3. HCV NS2 Associates with Glycoproteins E1 and E2 through Direct Protein–Protein Interactions

After cell lysis in Tx or TxnO buffer, NS2 was still co-immunoprecipitated with AR3A (anti-E2) or A4 (anti-E1), although the signal was weaker for co-immunoprecipitation with A4 (Figure 2B,D,E). By contrast, NS2 was only detected after cell lysis in Tx buffer (not in TxF and TxnO buffers) when AR5A was used (Figure 2C). Thus, as DRMs were completely solubilized by TxnO buffer, NS2 probably interacts with E1 and the E2 species recognized by AR3A through direct protein–protein interactions. By contrast, after DRM disruption, NS2 associates only very weakly, if at all, with the native E1E2 complex (targeted by AR5A).

### 3.4. HCV NS2 Associates with NS3 and NS5A through DRMs

We investigated whether the co-immunoprecipitation of HCV NS3 and NS5A with NS2 described in previous studies [6,8,9] involved DRMs or direct protein–protein interactions (as shown for E1 and E2, the targets of A4 and AR3A, respectively).

When Tx buffer was used, NS2 was co-immunoprecipitated with the HCV glycoproteins NS3 and NS5A together (Figure 2C), as previously demonstrated [6,8,9]. HCV NS2 and E2 (AR3A-E2) co-immunoprecipitated NS3 after lysis with Tx or TxF buffer (Figure 2B,C). By contrast, no co-immunoprecipitation was observed when DRMs were solubilized (TxnO buffer) (Figure 2B,C). With AR5A, NS3 was co-immunoprecipitated only when Tx buffer was used (Figure 2C).

We detected no co-immunoprecipitation of NS3 with the anti-E1 Ab A4 (contrasting with the results for NS2) (Figure 2D). Although NS3 interacts with E2 after Tx buffer lysis, and E1 associates with E2 via the E1E2 complex, A4 recognizes all E1 proteins translated by the cell machinery (unlike AR3A, which recognizes a conformational epitope on E2 proteins). Thus, A4 immunoprecipitates all E1 proteins (monomeric or involved in the E1E2 complex, whether associated with DRM or not), rendering E1-NS3 co-immunoprecipitation a minor, undetectable event. In addition, when Tx buffer was used, NS5A was co-immunoprecipitated with E2 (AR3A-E2) and NS2 (Figure 2B,C). After TxF lysis, NS5A was no longer co-immunoprecipitated with these two proteins (Figure 2B,C). No NS5A co-precipitation was detected with AR5A or A4 (Figure 2C,D).

Thus, NS2 interacts with E1 and AR3A-E2 species through direct protein–protein interactions, and with NS3 through DRMs. NS5A was found to be associated with E2 and NS2 via DRMs, as these interactions were detected only when Tx buffer was used. These interactions were abolished in TxnO and, surprisingly, TxF conditions (Figure 2B,C). The freeze–thaw cycle may have disrupted weaker parts of these DRMs, destabilizing the interactions of NS5A with other HCV proteins. Thus, HCV NS5A is also associated with NS2, NS3, E1, and AR3A-E2 through unstable DRM regions (uDRMs). These unstable domains may correspond to the boundaries of DRMs or the interface. By contrast, the complex formed by NS2, E1, AR3A-E2, and NS3 was preserved after the freeze–thaw cycle (Figure 2B,C). We detected no interaction between the native E1E2 complex (AR5A target) and NS5A.

### 3.5. The Native E1E2 Complex Associates Efficiently with ApoE, but Much Less Efficiently with NS2 and NS3

**Figure 3:**
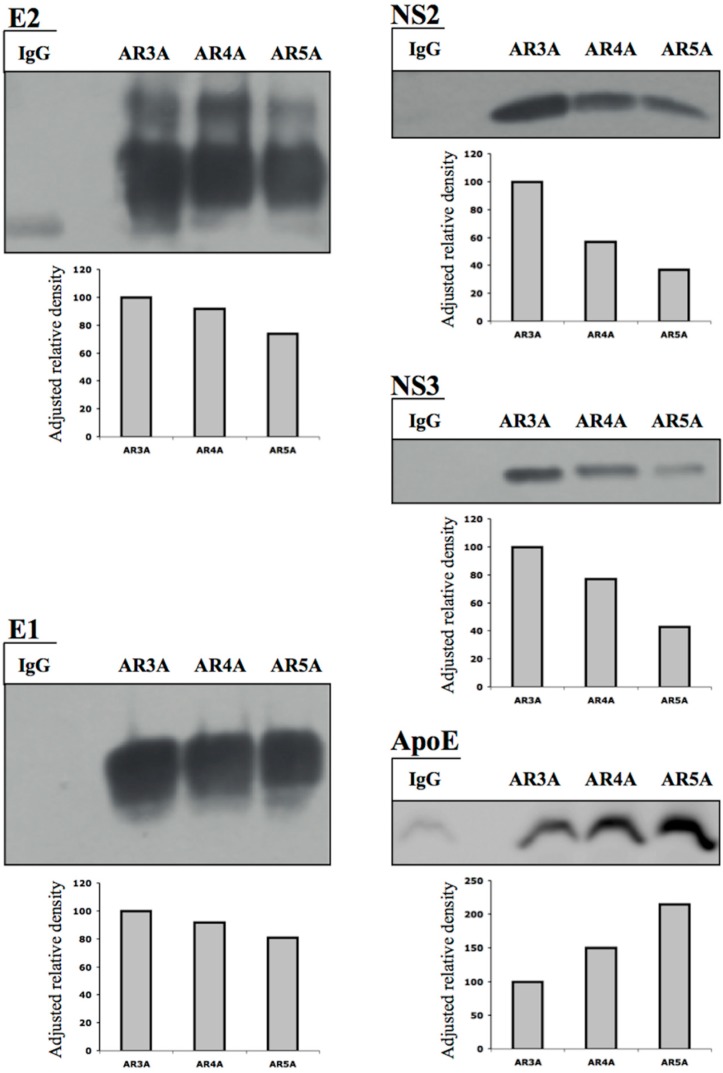
HCV E1E2 folding determines the efficiency of association with viral and cellular proteins. ^A4HA^HCVcc-infected cells were lysed with Triton X-100 (Tx) buffer. The resulting cell lysate was subjected to immunoprecipitation with anti-E2 (AR3A), anti-E1E2 (AR4A), and anti-E1E2 (AR5A) antibodies, and with isotypic IgG. The immunoprecipitated proteins were subjected to immunoblotting with anti-E2, anti-E1, anti-NS3, and anti-HA antibodies. Protein bands were quantified by densitometry using ImageJ software. AR3A immunoprecipitation band relative density is 100. The western blots presented here are representative of multiple experiments (≥3) showing similar results.

The anti-E1E2 Abs AR4A and AR5A are specific for well-folded native E1E2 complexes, the protein conformation associated with infectious particles, but they have different epitopes and apparent affinities for E1E2 [21]. We previously showed that AR5A was more efficient than AR3A and AR4A for ApoE co-immunoprecipitation from the lysates of infected cells [27]. These results were confirmed in this study (Figure 3). By contrast, we show here that the amounts of NS3 and NS2 co-immunoprecipitated with E2 were largest with AR3A, followed by AR4A and AR5A (Figure 3). These data suggest that the E2 species targeted by AR3A (mostly intermediate folding species) associates most efficiently with NS2 and NS3, as the association was much weaker when AR5A was used (Figure 2C and Figure 3). By contrast, E1E2 complexes (AR5A-E1E2) associated robustly with ApoE, whereas co-immunoprecipitation with AR3A-E2 was less efficient (Figure 3). Our results suggest the existence of a regulated protein coalescence process in which the HCV envelope protein species interacting with NS2 and ApoE are different. This mechanism would allow the NS2-driven collection of envelope proteins at the assembly site without impairing subsequent association of the native E1E2 form with ApoE and nascent particles.

Small amounts of the NS2 and NS3 proteins were co-immunoprecipitated with the E1E2 complex (AR5A-E1E2) after lysis in Tx buffer. After lysis in Tx buffer plus a freeze–thaw cycle, neither NS3 nor NS2 was co-immunoprecipitated with AR5A-E1E2 (Figure 2C). These data suggest that, like NS5A, the E1E2 complex is associated with NS3 and NS2 through a less stable DRM region. By contrast, after TxnO lysis, NS2 was co-immunoprecipitated with E1 and AR3A-E2 (Figure 2B,C). These results suggest that the degree of E1/E2 folding may determine the nature (protein–protein or through DRMs) and efficiency of envelope protein interactions with viral and cellular proteins (NS2, NS3, NS5A, ApoE).

### 3.6. HCV Core Associates with NS2, Well-Folded E2, and the E1E2 Complex through Less Stable Domains of DRMs

**Figure 4:**
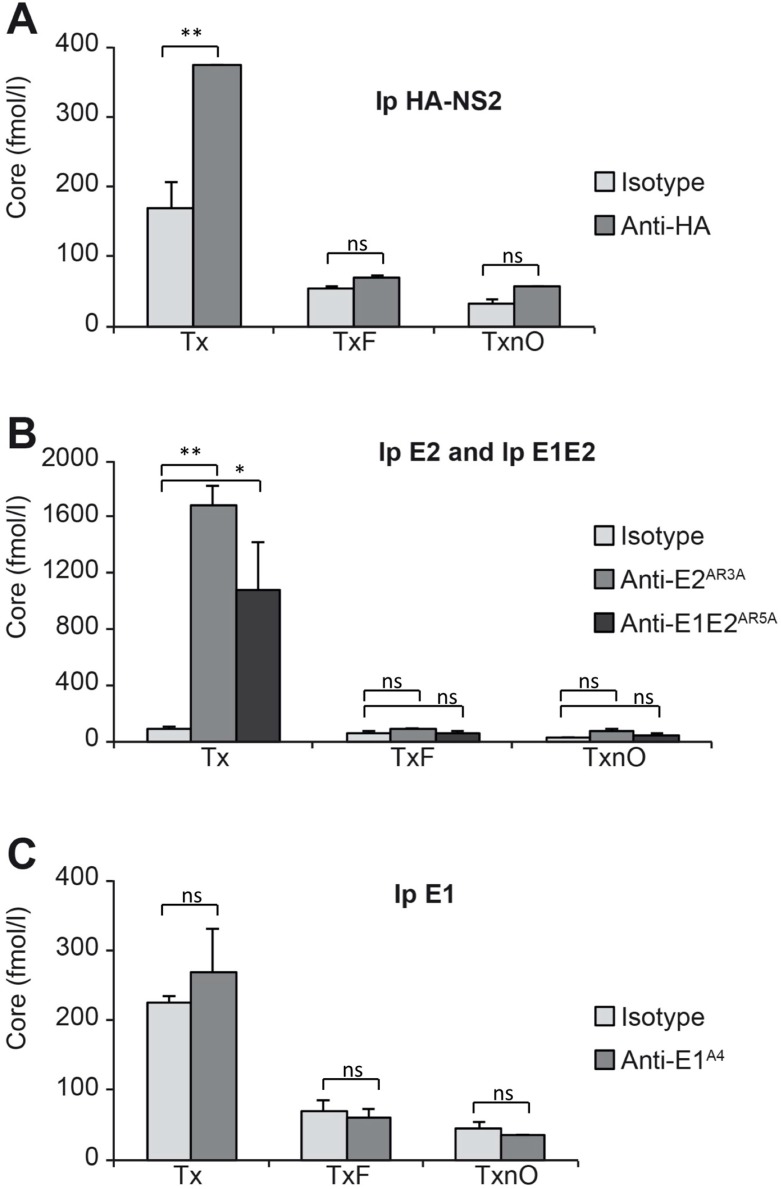
Involvement of DRMs in the association of HCV core with HCV NS2 and the E1E2 complex. ^A4HA^HCVcc-infected cells were lysed with Triton X-100 (Tx) buffer or Triton X-100 buffer + freezing (TxF) or Triton X-100 buffer + freezing + n-octyl-β-d-glucopyranoside buffer (TxnO). Core was quantified by a fully automated microparticle chemiluminescence immunoassay after immunoprecipitation (Ip) from cell lysates with (**A**) anti-HA, (**B**) anti-E2 (AR3A), anti-E1E2, (AR5A), (**C**) anti-E1 (A4) antibodies, and isotypic IgG. ns (not statistically significant), *p* > 0.05; * *p* < 0.05; ** *p* < 0.01.

Previous studies showed an absence of HCV core co-immunoprecipitation with NS2 [6,7,8,9]. We investigated whether a more sensitive technology could detect small amounts of core co-immunoprecipitated with NS2, E1, AR3A-E2, and well-folded E1E2 complex, by quantifying the co-precipitated HCV core protein in a fully automated microparticle chemiluminescence immunoassay (described in Materials and Methods) (Figure 4). When Tx buffer was used, we detected significant co-immunoprecipitation of core with NS2, AR3A-E2, and AR5A-E1E2 (Figure 4A,B). After lysis in TxF or TxnO buffer, core was no longer co-immunoprecipitated with NS2, AR3A-E2, or E1E2 (Figure 4A,B). By contrast, in experiments with anti-E1 antibody (A4), no co-immunoprecipitation of core was observed (Figure 4C).

We show here that core is also associated with NS2, AR3A-E2, and AR5A-E1E2 through DRMs. This association can be disrupted by a single freeze–thaw cycle. We therefore suggest that core associates with the NS2-driven protein complex (NS3-E1-E2-NS5A) through less stable domains of DRM. Core and the native E1E2 complex have this property in common: NS2 may drive these protein species to the assembly site, but remains loosely associated with these proteins.

### 3.7. HCV NS2, E2, NS5A and the E1E2 Complex Are Colocalized at the Same Membranes of the Membranous Web

**Figure 5:**
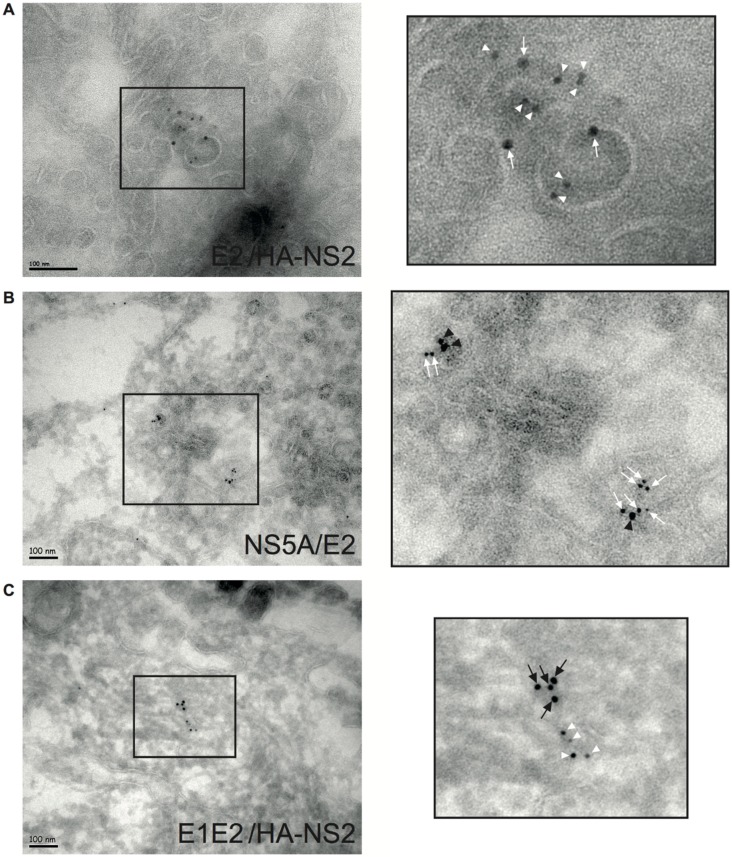
Colocalization of HCV E2 glycoprotein, HCV E1E2 complex, NS2, and NS5A at the same ER membranes from the membranous web. ^A4HA^HCVcc-infected cells were processed for electron cryomicroscopy double-labeling (**A**) E2 (AR3A + 10 nm gold-secondary Ab) and HA-NS2 (anti-HA + 6 nm gold-secondary Ab), (**B**) NS5A (9E10 + 10 nm gold-secondary Ab) and E2 (AR3A + 6 nm gold-secondary Ab), and (**C**) E1E2 complex (AR5A + 10 nm gold-secondary Ab) and HA-NS2 (anti-HA + 6 nm gold-secondary Ab). These representative images show the membranous web with E2 (white arrow), NS2 (white arrowhead), NS5A (black arrowhead), and E1E2 complex (black arrow) lying on the ER bilayer. The images in the column on the right are enlargements of the areas within the squares.

We checked for potential specific intracellular structures accommodating E1E2, NS2, and NS5A at the same site by examining immunolabeled cryosections of infected cells under a transmission electron microscope (as described in the Materials and Methods section). Our goal was to determine whether DRMs carrying these proteins could be visualized by transmission electron microscopy. Various cryosections were immunogold-labeled with anti-HA (NS2), anti-NS5A, AR3A, or AR5A antibodies. The labeled proteins were found almost exclusively on vesicle membranes reminiscent of the vesicles constituting the membranous web (Figure 5). The membranous web is a specific alteration of the ER membranes, induced by HCV proteins and used by the virus as a scaffold for the HCV RC [28]. We found that the AR3A-E2 and NS2 proteins (Figure 5A) were almost always located on the same vesicle membrane, in close proximity. A similar distribution was observed for AR3A-E2 and NS5A (Figure 5B).

In addition, the AR5A-E1E2 complex and the NS2 protein were localized to the same membrane, but were always found in two different areas (Figure 5C). This observation is consistent with our previous results showing the efficient co-immunoprecipitation of NS2 with AR3A-E2, and the loose association of NS2 with the native E1E2 complex. DRM-like structures were never clearly visualized in our preparations.

Nevertheless, collectively, these observations support our immunoprecipitation data (Figure 2 and Figure 5). NS2, E1, and AR3A-E2 interact directly, whereas NS2 interacts with NS3 and NS5A through DRMs. The well-folded E1E2 complex interacts with the NS2 complex through a less stable domain of DRM, and may be the envelope species involved in the budding step.

## 4. Discussion

Recent studies have described the formation of protein complexes at the assembly site, under the control of NS2. This nonstructural protein initiates virus assembly by coordinating interaction between glycoproteins E1, E2, NS3-4A, and NS5A [5,6,7,8,9]. In the resulting complex, NS3 is associated with NS4A [9], because NS4A stabilizes NS3 and anchors this protein in the ER membrane [29]. It has been suggested that NC translocation into the ER is initiated immediately after the NS2-driven relocalization of the proteins described above. Consequently, understanding the mechanism by which NS2 promotes the initiation of HCV morphogenesis may facilitate identification of the HCV assembly platform. Our observations demonstrate the key role of DRMs (or lipid-raft-like structures) in bringing the proteins required for virus assembly together. Our results confirm the findings reported for NS2 in previous studies [6,7,8,9], and highlight the existence of a protein coalescence process organized by NS2 around DRMs. Interestingly, DRMs have been shown to interact with viral proteins and to stabilize RC, and they may be an integral part of infectious HCV particles [11,13,14,15,16]. They also facilitate virus assembly [30]. These findings are consistent with a major role for lipid-raft-like structures in bringing together the proteins involved in the initiation of viral morphogenesis.

**Figure 6:**
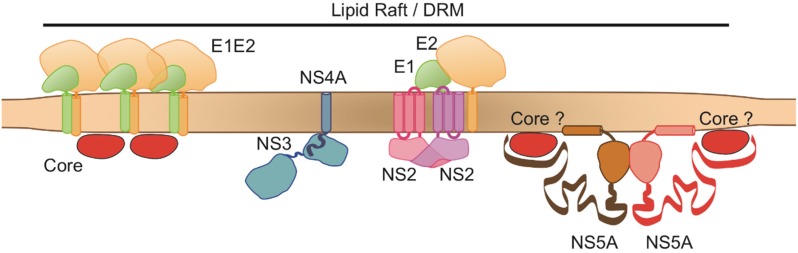
Model of the interactions of HCV proteins and their localization at the proposed viral assembly platform. Our experiments characterized associations of HCV proteins with two different DRM subdomains: a stable domain (Brown region) associated with NS2, NS3 (NS4A), AR3A-E2, and A4-E1, and a less stable domain (light brown region) accommodating NS5A, core, and AR5A-E1E2. One of the first steps in the process is the recruitment by NS2 of glycoproteins (E1, recognized by A4, and E2, recognized by AR3A) to stable, raft-like structures through direct protein–protein interactions. NS2 simultaneously recruits NS3-4A to these same structures through an indirect DRM-driven interaction. NS5A as well as core associate with NS2 through unstable, possibly outlying, regions of DRMs.

We have shown that the interaction between the NS2 and HCV proteins occurs directly (protein–protein interactions) or through DRMs (Figure 6). We suggest here that DRMs are heterogeneous in terms of structure and function: one DRM domain (possibly the central part) is particularly robust (stable DRMs, sDRMs), and solubilized only with our strong TxnO lysis buffer. One unique property of TxnO lysis buffer is that it prevents protein aggregation and protein denaturation [24]. Outside this “main domain” (possibly at the boundaries or interface), the DRM may be less stable and can be solubilized with Tx buffer plus a freeze–thaw cycle (unstable DRMs, uDRMs). These different regions may have different concentrations of cholesterol and sphingolipids, which would also differ from those of ER membranes. We have shown that native AR5A-E1E2 complexes and A4-E1/AR3A-E2 proteins have their own separate functions and are probably involved in different steps in the process.

We have demonstrated that the E1E2 complex is associated with the complex formed by NS2, NS3, E1, E2, and core, via the unstable domains of DRMs (Figure 6). Our data suggest that a complex mechanism of HCV protein recruitment is involved in budding, with NS2 acting as a protein complex organizer around lipid-raft-like structures (DRMs). We suggest that one of the first steps in the process is the recruitment, by NS2, of glycoproteins (E1, recognized by A4, and E2, recognized by AR3A) to these lipid-raft-like structures through direct protein–protein interactions (Figure 6).

NS2 simultaneously recruits NS3-4A to these structures through an indirect, DRM-driven interaction. NS3-4A may then mobilize HCV RNA from RC to DRMs through p7 [11], although this protein does not seem to be present in DRMs [12]. Tedbury et al. showed that, following disruption of the localization of NS2 to DRMs, this protein was no longer co-immunoprecipitated with NS5A. They did not detect HCV NS5A in insoluble fractions, highlighting the dependence of NS2 association with NS5A on DRMs [11]. Our data partly confirm these observations, as the partial disruption of DRMs resulted in a lack of association of NS5A with NS2. Our findings thus indicate that NS5A associates with NS2 through unstable, possibly outlying, regions of DRMs. Interestingly, the interaction of NS5A with core initiates the association of core with RNA to form the NC [31]. Our results indicate that core associates with NS2 through DRMs. Popescu et al. suggested that the association of NS5A with the p7-NS2-E1-E2 complex is the second step in complex formation, initiating the assembly process [8]. Thus, before translocation of the NC into the ER, the NS2-E1-E2-NS3-4A-RNA complex forms in the DRMs. NS5A may then also associate with the DRMs, but only in unstable outlying regions (uDRMs). Core may be mobilized by NS5A at this stage.

We also demonstrate that, in parallel, well-folded E1E2 complexes interact with NS2, NS3, and core, but not with NS5A (Figure 2 and Figure 4). We previously showed that native E1E2 complexes also interact with ApoE at the initiation of HCV morphogenesis. This interaction between native E1E2 and ApoE may trigger the budding of NC into ER lumen [27]. This dual interaction mediated by envelope proteins across the ER membrane (interaction of envelope proteins with ApoE and NS2) may involve two different glycoprotein species. AR3A-E2 interacts efficiently with NS2, and the presence of a well-folded native complex is dispensable for this interaction. E2 proteins (not necessarily involved in native complexes) are the major species found in infected cells. The second glycoprotein species, the native E1E2 found on mature HCV particles, is the glycoprotein species efficiently associated with ApoE. It is possible that AR3A-E2, which is produced in large amounts, interacts with a large number of proteins, including, specifically, NS2, efficiently initiating the process, whereas the E1E2 complex later activates the budding into the ER of particles carrying mature E1E2 proteins by interacting with ApoE.

There are several lines of evidence to suggest that DRMs are involved in this budding initiation step. Indeed, the modified ER membranes accommodating the RC contain nine times as much cholesterol as ER membranes in general [15]. This concentration of cholesterol is similar to that reported in DRMs. The production of HCV particles is dependent on sphingolipid biosynthesis, whereas genome replication is not [14]. Cholesterol and sphingolipids, two of the main components of DRMs, are also detected in viral particles. These molecules play a key role in the internalization of viruses by cells [14].

Thus, lipid-raft-like structures seem to be crucial for many steps in the HCV life cycle, and, according to the findings presented here, may play a key role in initiating particle morphogenesis.

In summary, we suggest that the coalescence of HCV proteins organized by NS2 occurs on lipid-raft-like structures through direct (E1 and AR3A-E2) and indirect (supported by a common association with DRMs) interactions. Our data suggest the existence of a regulated mechanism organized by NS2 around DRMs: NS3 is involved in the initial interaction, whereas NS5A may associate with lipid-raft-like structures during a subsequent step, and with outlying unstable regions of DRMs. Once associated with DRMs, NS5A may mobilize HCV core to the assembly site. These interactions take place in a step immediately preceding NC translocation into the ER. We therefore suggest that DRMs carrying the NS2-driven protein complex may act as an assembly platform for HCV (Figure 6). Translocation may then be triggered by an interaction of the native E1E2 protein complex with apolipoproteins, such as ApoE, in particular.

We do not expect translational results of this research.
